# Primary Cutaneous Adenoid Cystic Carcinoma of the Back: A Case Report

**DOI:** 10.7759/cureus.49099

**Published:** 2023-11-20

**Authors:** Aimee H Dubin, Qwynton Johnson, Joel Horowitz

**Affiliations:** 1 Surgery, Campbell University School of Osteopathic Medicine, Lillington, USA; 2 Surgery, Kansas City University, Kansas City, USA; 3 Surgery, Cape Fear Valley Medical Center, Fayetteville, USA

**Keywords:** perineural invasion, radiation therapy, epidermal inclusion cyst, adenoid cystic carcinoma, skin cancer

## Abstract

Adenoid cystic carcinoma (ACC) is a rare type of carcinoma that arises from the salivary glands. When ACC is present on the skin with no other primary site of malignancy in the body, it is termed primary cutaneous adenoid cystic carcinoma (PCACC). The only way to differentiate between ACC and other benign cutaneous masses is through the use of histopathology and immunohistochemistry. This case report describes a 67-year-old Asian female with a history of an epidermal inclusion cyst. She was seen in consultation with general surgery for the removal of a mass on her lower back. The initial excision's pathology revealed an ACC with perineural invasion. However, there were positive margins, as the mass was originally thought to be benign. Consequently, she underwent a second procedure for the total excision of the mass, resulting in subsequent negative margins. The patient was referred to radiation oncology; however, she ultimately opted to defer postoperative adjuvant radiation therapy, with the understanding that she would undergo biannual screening examinations.

## Introduction

Obtaining an accurate incidence of soft tissue tumors poses a significant challenge, primarily due to the nature of the condition. For instance, soft tissue masses can often go unnoticed by the individual or may not warrant medical attention [1]. Epidermal inclusion cysts are one of the most common benign soft tissue masses and can be found anywhere on the body [2]. They typically manifest during the third and fourth decades of life, with the mainstay of treatment being complete surgical excision of both the cyst and its surrounding wall [3]. Similarly to epidermoid cysts, lipomas are also common benign soft tissue neoplasms. They are found in one percent of the population and tend to arise in individuals aged 40 to 60, with no gender preference [4]. Although rare, lipomas can potentially transform malignant into liposarcomas [5, 6]. Both epidermoid cysts and lipomas often share a similar presentation, with typically no associated pain. As a result, it is difficult to differentiate malignant conditions from their benign counterparts. To try to circumvent this issue, clinicians must maintain a high level of suspicion when assessing for potential malignancies, as many develop indolent in nature. The utilization of various diagnostic modalities, including imaging, pathology, and immunohistochemistry, plays a crucial role in accurately diagnosing seemingly benign conditions. Adenoid cystic carcinoma (ACC) originates from salivary glands and is a malignant, slow-growing disease. Additionally, it presents features identical to epidermal inclusion cysts. Although occurring primarily in glandular tissue, ACC has also been observed in various other locations, including the breast, uterus, cervix, lacrimal glands, and bronchi [6, 7]. In rare instances, ACC can present as a primary cutaneous lesion, known as primary cutaneous adenoid cystic carcinoma (PCACC). It is impossible to tell ACC and PCACC apart using histopathology alone. As a result, the only way to differentiate the two carcinomas is to determine where the primary lesion lies through thorough imaging and testing for distant metastases [8, 9]. Microscopically, another differentiating factor of PCACC from ACC is that it is a dermal-based tumor, meaning it lacks a connection to the epidermis. In this case, we highlight the need for histopathological examination to identify rare malignant dermatologic conditions.

## Case presentation

**Figure 1:**
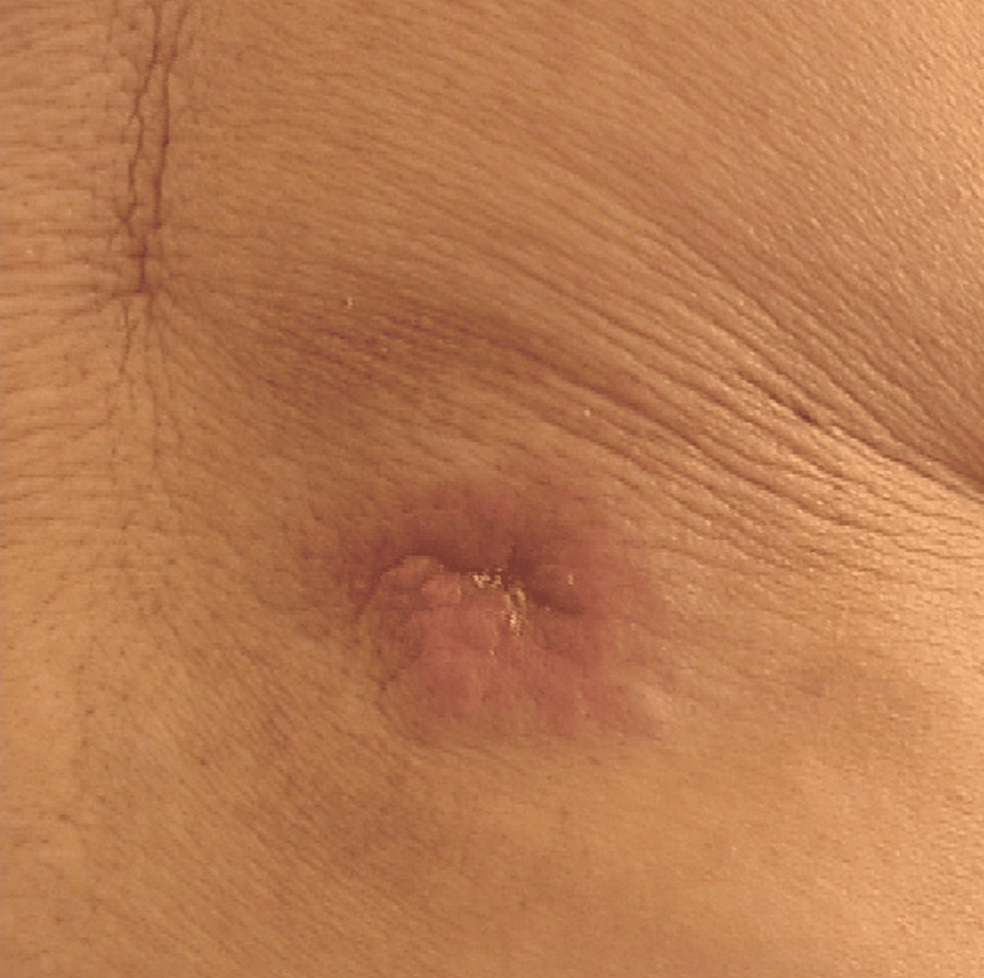
A 3-cm non-erythematous firm mobile nodule. The first surgical excision revealed positive margins. Upon wide local re-excision, the lesion measured nine centimeters from medial to lateral, and four centimeters from superior to inferior, with a depth of one and a half centimeters. The skin surface appeared pink-brown and smooth. The specimen was serially sectioned from lateral to medial. This image is representative, as one was not taken before surgery [10].

**Figure 2:**
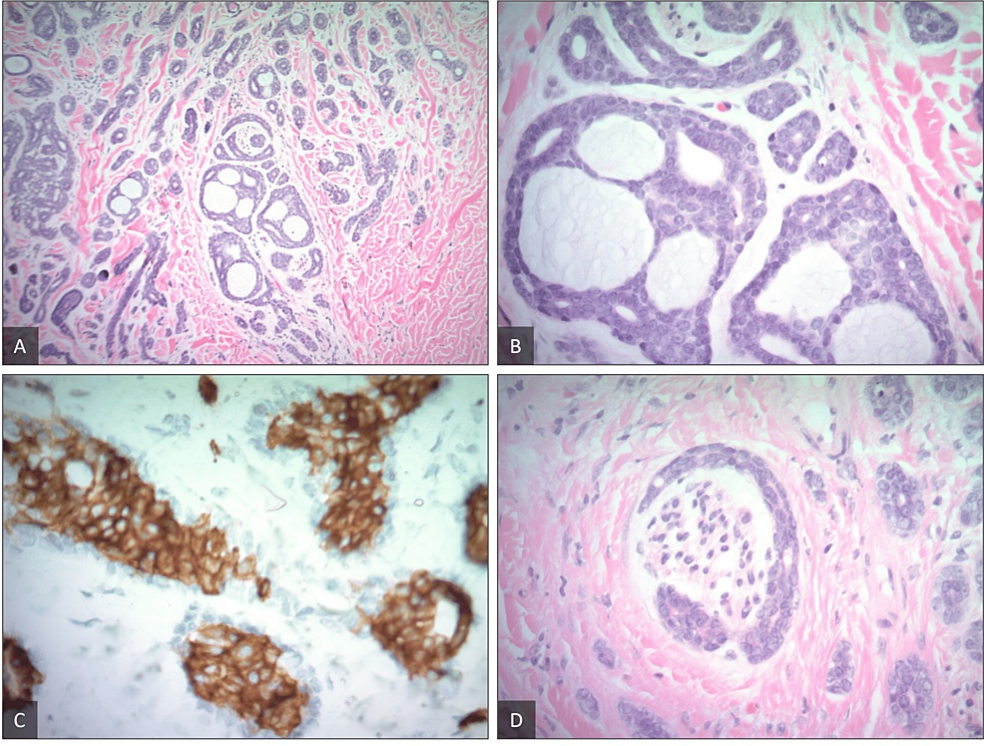
Histopathology of specimen. Histopathologic images were taken from the excised specimen. (A) H&E at 10x magnification; (B) H&E at 40x magnification. Both magnifications showed evidence of dermal epithelial proliferation without connection to the epidermis, as well as nests of basaloid cells with tubular and cribriform morphology. Occasional mitotic figures are present. (C) Immunohistochemistry (IHC) demonstrated diffuse, strong positive reactivity for CD117 in the tumor cells. (D) The images also show evidence of perineural invasion.

A postmenopausal 67-year-old Asian female with a past medical history of a prior epidermal inclusion cyst removal was referred to a general surgeon for the excision of a nodule on her lower back. At the time of the referral, her medication list included cyclosporine ophthalmic drops, fexofenadine, citalopram, and atorvastatin. Her surgical history included an appendectomy, an epidermal cyst removal, and a hysterectomy. Notably, the patient endorsed a 50-year smoking history. Initially, the patient noticed a painless 3 cm nodule on her lower back, which gradually became firmer over a span of several months. The presentation of the nodule closely resembled her previous epidermal inclusion cyst (Figure 1). Consequently, based on her presentation and initial consultation, she was scheduled for surgical excision of the mass. After excision, the pathology report (Figure 2A-2D) revealed a 3 cm ACC of the right lower back. The tumor was 7 mm thick and was noted to extend into the subcutaneous adipose tissue with evidence of perineural invasion. Following excision, pathological findings indicated that the lesion was present extensively at the lateral margins. Additionally, the tissue exhibited dermal epithelial proliferation without connection to the epidermis and consisted of nests of basaloid cells with tubular and cribriform morphology. Lastly, the ductal component of the tumor showed strong positive reactivity for CD117, and the myoepithelial component of the tumor showed positive expression of S100.

After the initial pathology report, the patient was recommended to undergo wide excision with potential postoperative radiation therapy. Approximately one month after the initial excision, she underwent wide excision with subsequent negative margins. Subsequently, a contrast-enhanced CT of the abdomen, chest, and pelvis was performed, which revealed no evidence of metastatic disease. Following the second procedure, the patient was evaluated by a radiation oncologist. Given that histological findings were consistent with ACC, exceeding two cm with the presence of perineural invasion, radiation therapy was strongly advised as the standard of care. The oncologist recommended a 50 Gray (gy) radiation regimen delivered in 20 fractions. However, the patient ultimately deferred postoperative radiation therapy due to a lack of evidence of metastatic disease. Eight months following her initial visit with general surgery, the patient denied any new symptoms, pain from the incision site, unintended weight loss, or signs of fatigue. Her physical exam was unremarkable, with a well-healed 10 cm incision and no evidence of local recurrence on her lower right back. She expressed no interest in postoperative adjuvant radiation and was therefore advised to follow-up every six months for repeat examinations.

## Discussion

PCACC most commonly presents on the face, head, and neck, with the highest incidence occurring on the head. The incidence decreases as the lesion moves more distal from the head, with the extremities being the least likely area to develop PCACC [11]. Guidelines for treatment remain unclear, with only 250 cases reported in the literature. However, recommendations of two cm wide local surgical excision and potential adjuvant chemo-radiation therapy to minimize recurrence have been shown to be effective [12]. Although metastasis of PCACC is rare, perineural involvement increases the tendency for local recurrence, which occurs 76% of the time [13]. Assessing the extent of perineural invasion proves to be difficult, which is why prompt adjuvant therapy or sentinel lymph node biopsies are often indicated [14-16]. The use of postoperative chemotherapeutic agents or radiation is controversial, with conflicting opinions among experts. However, in patients with negative margins and continuous benign follow-up, the most common consensus is to avoid chemo-radiation due to an increased association with developing lymphohematopoietic cancers post PCACC excision [11, 13, 17]. Histological evaluation is the mainstay for distinguishing PCACC from other metastatic cancers or benign cysts. For instance, this case demonstrates PCACC being potentially disguised as an epidermal inclusion cyst due to the patient's prior history of having a benign mass removed. However, histology was the only way to determine that this was a malignant neoplasm that prompted further surgical excision for negative margins and a thorough evaluation of potential metastasis. Perineural involvement is a characteristic feature of PCACC. Additionally, various histopathological markers are indicative of this tumor type. Specifically, mucin secretion is typically observed, along with two distinct cell populations: ductal and myoepithelial [14]. These cell types are positive for CD117 and S100, respectively. These markers are crucial as they are not present in other types of cutaneous carcinomas, such as adenoid basal cell carcinoma. This underscores the importance of cytological pathology and immunohistochemistry in guiding the diagnosis and treatment of such tumors [14].

## Conclusions

PCACC is a rare, locally aggressive phenomenon with the potential to metastasize. It is seldom described in the literature due to its limited presentation in the population and the resulting lack of knowledge regarding it. Due to these reasons, PCACC can easily be mistaken for a benign process such as a lipoma or an epidermal inclusion cyst. This case report highlights the importance of expert histopathological evaluation of tissue specimens in order to allow for an appropriate diagnosis and management of rare disease processes.
